# Bacteriophages in Natural and Artificial Environments

**DOI:** 10.3390/pathogens8030100

**Published:** 2019-07-12

**Authors:** Steven Batinovic, Flavia Wassef, Sarah A. Knowler, Daniel T.F. Rice, Cassandra R. Stanton, Jayson Rose, Joseph Tucci, Tadashi Nittami, Antony Vinh, Grant R. Drummond, Christopher G. Sobey, Hiu Tat Chan, Robert J. Seviour, Steve Petrovski, Ashley E. Franks

**Affiliations:** 1 Department of Physiology, Anatomy & Microbiology, La Trobe University, Bundoora, VIC 3086, Australia; 2 Department of Pharmacy & Biomedical Sciences, La Trobe University, Bendigo, VIC 3550, Australia; 3 Division of Materials Science and Chemical Engineering, Yokohama National University, Yokohama 240-8501, Japan

**Keywords:** bacteriophage, environment, human body, phage therapy, phage biocontrol, soil, water, wastewater, pharmaceutical products

## Abstract

Bacteriophages (phages) are biological entities that have attracted a great deal of attention in recent years. They have been reported as the most abundant biological entities on the planet and their ability to impact the composition of bacterial communities is of great interest. In this review, we aim to explore where phages exist in natural and artificial environments and how they impact communities. The natural environment in this review will focus on the human body, soils, and the marine environment. In these naturally occurring environments there is an abundance of phages suggesting a role in the maintenance of bacterial community homeostasis. The artificial environment focuses on wastewater treatment plants, industrial processes, followed by pharmaceutical formulations. As in natural environments, the existence of bacteria in manmade wastewater treatment plants and industrial processes inevitably attracts phages. The presence of phages in these environments can inhibit the bacteria required for efficient water treatment or food production. Alternatively, they can have a positive impact by eliminating recalcitrant organisms. Finally, we conclude by describing how phages can be manipulated or formulated into pharmaceutical products in the laboratory for use in natural or artificial environments.

## 1. Introduction

With advances in DNA sequencing and genomics over the last decade, microbiome research has increased at an exponential rate [1]. The 16S ribosomal RNA gene sequencing data generated have aided our understanding of natural bacterial community biodiversity. Metagenomic studies with complex communities have elucidated additional genome sequences, not only from prokaryotes but also eukaryotes and viruses [1]. However, understanding the ecological implications of such data can be highly challenging [1]. A microbial community can work together in mutualistic and antagonistic relationships to perform a defined function, but this may be disrupted if the stability of that community changes. For example, change to the composition of the microbiome in the human body is known to impact on other biological functions, including the immune system and mood [2,3]. Such shifts in community balance can be responses to a range of both exogenous and endogenous parameters, including exposure to bacteriophage (or phage). It is estimated that there are 10^31^ phage particles on earth, ten-fold more than bacterial population estimates, making phages the most abundant biological entities in the biosphere [4].

Phages infect their host by a range of biochemically diverse host surface receptors, such as carbohydrates, lipopolysaccharides, and proteins [5,6]. The phage host range is generally determined by how specifically the phage interacts with the host receptor, such that recognition of a highly unique region can lead to a narrow host range where a phage may be capable of infecting only a single host species or strain [5,6].

**Figure 1:**
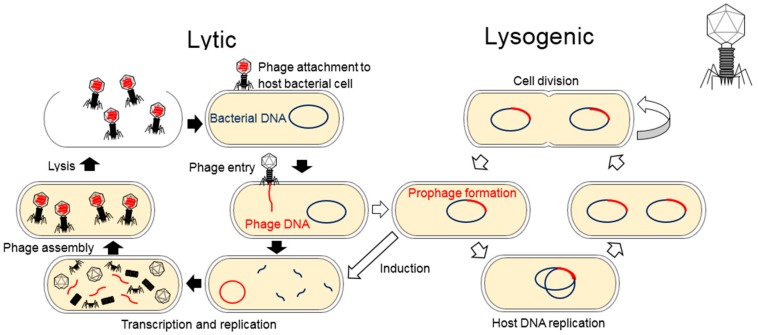
Bacteriophage Lifecycle: Lytic phages attach and infect a bacterial cell which results in the reproduction of phages and lysis of the cell host and this lysogenic cycle results in the integration of a phage genome into the bacterial genome. Some lysogenic phages do not integrate into the genome and remain in the cell as a circular or linear plasmid (not depicted here).

Phage life cycles are categorized into two groups which are lytic and temperate. When the lytic phage genome is injected into its host, it initiates processes that hijack the host metabolic systems to ultimately produce multiple viral progenies, which are then released from the host utilizing phage encoded lytic enzymes. These progenies are able to infect new hosts and repeat the lytic cycle (Figure 1). Temperate phage infection may lead to the aforementioned lytic cycle, or alternatively a lysogenic lifecycle, where the phage becomes a prophage by integrating its genetic material into the host genome or forms either circular or linear plasmid within the host cytoplasm. The prophage replicates in synchrony with the host genome until its lytic cycle is induced (Figure 1) [7]. Prophage integration often provides immunity from a superinfection, which is a secondary infection of phages, due to the expression of phage resistance genes by the prophage [8]. This resistance arises through several mechanisms including prophage mediated changes in cell surface receptors which prevents subsequent phage attachment [9]. This immunity can protect a host against phages that are close (homotypic defense) or distant relatives (heterotypic defense) [10].

**Figure 2:**
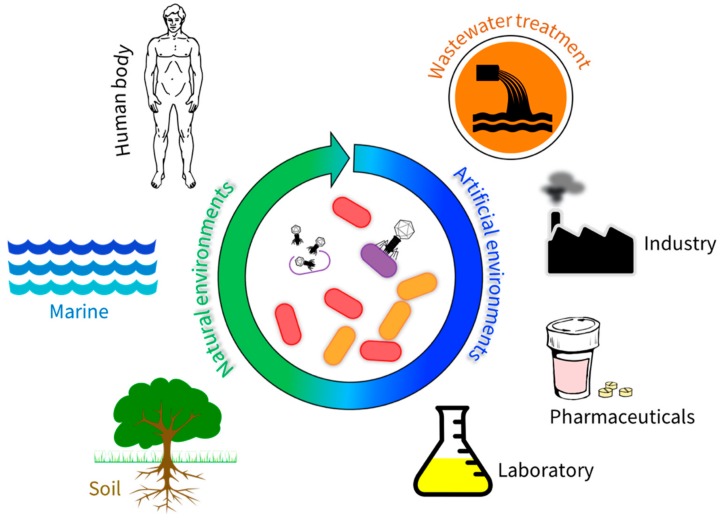
Bacteriophages in natural and artificial environments: The significance of phages in natural environments lies in their ability to replicate within their host, thus impacting the diversity of bacterial communities. Within the human body, phages function to protect against pathogenic bacteria. Ocean waters contain 4 × 10^30^ viruses, making it the largest phage reservoir. Soil phages influence nutrient cycling capabilities and symbioses between plant roots and bacteria. In artificial environments, the ability of phages to mediate bacterial growth can be exploited for a range of uses, due to their specificity and how readily they can be genetically modified. In wastewater treatment, phages can be used to impact the bacterial communities present, thus increasing the efficiency of this process. Industrial applications of phages include control of foodborne pathogens and decreasing the number of problematic bacteria in the petroleum industry. Pharmaceutical uses for phages are currently limited, but this is likely to change as the efficacy of antibiotics reduces and phage efficacy and specificity are improved in the laboratory.

Given the underlying flaws of lysogenic phages, in relation to acquisition of virulence factors and host immunity to superinfection, lytic phages present themselves as attractive candidates for phage therapy or biocontrol. Furthermore, lytic phages can markedly alter microbial communities and exploit their broader functions as this mini review will demonstrate. It will highlight the presence and role of phages in both natural and artificial environments and discuss important impacts phages have on their microbial communities (Figure 2). We show that the impact of lytic phages can be both beneficial and detrimental, which highlights the importance of studying phage communities as an integral part of microbial ecology.

## 2. Bacteriophages in Natural Environments

### 2.1. Bacteriophages Within the Human Body

The human body is colonized with over 10^12^ bacteria [11]. Most of these reside within the gut and have been the focus of host-microbiota research for the past decade [12,13]. Phages are also ubiquitous to body surfaces, including the skin, oral cavity, lungs, intestines, and urinary tract and they represent a natural predator to this extensive microbiome, outnumbering bacteria (by at least 10-fold) and play significant roles in shaping the composition of the bacterial communities within various bodily compartments [14,15,16,17]. Moreover, it has been demonstrated that phages can penetrate the epithelial lining of these structures via rapid directional transcytosis providing them with access to both vesicular and cytosolic compartments of eukaryotic cells [14,18,19,20]. It is estimated that 31 billion bacteriophage particles undergo transcytosis across the epithelial cells of the gut into the human body every day which means that there are ample opportunities for modulation of host cell signaling [14].

Mucosal surfaces are the main zones where microbes directly interact with their human hosts [21]. Hence, the mucus layers not only serve as the key points of entry for pathogenic microorganisms, but they are also heavily colonized by symbiotic bacteria that contribute additional genetic and metabolic potential to the host [21]. These resident symbionts benefit from increased nutrient availability, as well as the opportunity for both vertical transmission and increased dissemination [22,23,24,25,26]. Also residing within the mucus layer are bacteriophages, which can bind to mucin glycoproteins via immunoglobulin (Ig)-like domains in their capsids in a process termed “bacteriophage adherence to mucin” (BAM). BAM is thought to serve two important functions in the regulation of host–bacteria interactions. First, by accommodating bacteriophages with lytic activity, it provides the host with protection against pathogenic bacteria that might otherwise kill beneficial bacterial species or cause localized or systemic infections [27]. Indeed, there is evidence that, in response to pathogenic bacterial strains, the gut epithelium can actively modulate BAM and the composition of bacteriophages via hypersecretion of mucin and/or alteration of mucin glycosylation patterns in an effort to subvert microbial adherence and survival [28,29,30]. Second, mucus provides an environment for lysogenic bacteriophages to establish conditional symbiotic relationships with bacteria that are beneficial to their human hosts [31]. Integrated prophages frequently express genes that increase the fitness or virulence of their bacterial strain and protect them from infection of lytic phages. As free phages, they may also benefit their bacterial host strain by killing related bacteria that would otherwise represent competitors [32,33]. Indeed, studies have shown that intestinal commensal bacteria carrying prophage DNA produce infectious virions that limit interspecies competition [34,35]. Conversely, there is also evidence that under certain conditions (e.g., inflammatory stress) phage populations with lytic activity against beneficial members of the bacterial community are expanded, resulting in depletion of beneficial bacterial strains and expansion of pathogenic bacteria. The “dysbiosis” that results is potentially an important cause of acute and chronic illnesses [12,21,23,36,37].

Although phages are unable to directly infect eukaryotic cells, they, nonetheless, represent foreign bodies capable of triggering immune responses [38]. In some instances where phages are internalized into eukaryotic cells, they are uncoated, and their nucleic acids are released and have the potential to activate an immune response [39]. Toll-like receptors (TLRs) are one, among several, mechanisms where pathogen associated molecular patterns (PAMPs) are recognized [40]. In this context, TLR9, specifically, is able to recognize viral DNA after phagocytosis or transcytosis of phages [41]. When internalized, phage particles are either degraded or transported throughout the cell where transcription and translation of nucleic acids may occur and lead to cytokine production. It has been shown that there are positive effects of phage anti-inflammatory properties upon bacterial infection, however, this postulates that phages may heighten bacterial fitness and virulence by dampening immune responses [42]. Currently, the consequences of phage-mediated activation of immunity are unclear. However, it is conceivable that disruptions to what might be considered a “normal” phage population within a body compartment could lead to an inappropriate inflammatory response that gives rise to disease. Such disruptions might occur due to conditions associated with immunodeficiencies [43,44]; infections, dysbiosis or antibiotic treatments that cause lysis and release of phages from certain bacterial strains; and even changes in diet (e.g., switching from a low fat to a high fat diet) [45,46,47]. Indeed, emerging research has shown that inflammatory bowel disease (IBD) is associated with elevated levels of double-stranded DNA (dsDNA) phages and that such changes in the abundance and diversity of intestinal phages were independent of changes in the host bacterial communities [48,49,50]. This suggests that phages may directly contribute to the inflammation associated with IBD. Likewise, it was shown that changes to the composition of intestinal phage populations precede the development of type 1 diabetes in children [51]. This latter study further identified the specific phage strains that correlated with the onset of the disease as members of *Microviridae*, *Myoviridae*, and *Podoviridae* families and suggested there may be a phage component to the development of autoimmunity [51]. Finally, the administration of antibiotics induced transcription of genes associated with induction of lytic intestinal prophage replication, thus contributing to inflammation while also markedly altering both phage and bacterial intestinal communities [46,47].

It is also possible that changes in the phage population could have a beneficial effect on immune function by serving as a warning of an impending bacterial infection. Innate lymphoid cells (ILCs) are enriched at mucosal surfaces and employ fundamental defensive functions. The ILCs do not consist of antigen receptors, do not undergo clonal selection or expansion when stimulated, and are involved in repair responses in the presence of infection [52]. A seminal discovery showed that phages bind to TLRs via pathogen recognition and work in concert with neutrophils to promote efficient bacterial clearance during acute pneumonia [30]. Moreover, this study revealed that in the absence of neutrophils and myeloid cells, the immune system was unresponsive to phage treatment, thereby, prolonging infection which in turn suggested an “immunophage synergistic” relationship. Furthermore, phages have been shown to play a vital role in tumor regression via activation and recruitment of tumor-associated macrophages and neutrophils [53]. Similarly, antibiotic-resistant drugs have been an increasing threat to the treatment of infectious diseases. For example, the correlation of anomalous phage community composition to disease status suggests that phages could be utilized as biomarkers for the early detection of disease [54]. Studies have demonstrated that during the early onset of infection, phage treatment not only prevents bacterial invasion of epithelial cells, but also limits propagation and reduces immune cell recruitment [55]. If immune-modulating properties of phages can be validated, it is possible that phages could influence both host and bacterial interactions with each other and with the outcome of phage therapeutic interventions. The study of phage communities within the human microbiota is an emerging field, and much work is still needed to fully understand the impact of these resident viruses on health and disease. Indeed, such knowledge has the potential to unearth new biomarkers and therapeutics for a wide range of human diseases.

### 2.2. Bacteriophages in Marine Environments

Oceans are one of the most important and the largest environment on the planet, covering over 70% of the earth’s surface and providing approximately half of the world’s oxygen generation [56]. As a result, they encompass some of the most unique and complex ecosystems of any natural environment. This is also true of the microbes (including bacteria) of these communities which comprise 90% of the biomass of the ocean, dominating in terms of their abundance, diversity and metabolic function [57,58]. The viral portion of these communities is equally or more diverse and abundant [56]. It is estimated that phages are the most biologically abundant entities on the planet based largely on extrapolating the estimated numbers of viruses in our oceans. It is predicted ocean waters contain 4 × 10^30^ viruses in total, thus, outnumbering bacteria and archaea by more than 15-fold, with phages being the dominant viral group [56]. Because of this diversity and abundance, studies that are based around the virome of marine environments have been well documented and many recent reviews on phages in the surface layers of marine environments are available [56,57,59,60,61,62,63,64,65]. Some of their key findings are summarized here.

While studies have revealed phages to be the most abundant biological entities in oceans, taxonomic data is unavailable for approximately 60% of these, illustrating that little is still known about their diversity [66]. Among those for which taxonomic data is available, phages belonging to the order *Caudovirales* are the most abundant in surface layers of marine environments [67,68]. The order *Caudovirales* consists of double stranded DNA and tailed phages, which is split into three families, *Siphoviridae*, *Myoviridae*, and *Podoviridae* [69,70] based on differences in morphology (*Myoviridae* contain long contractile tails, *Siphoviridae* contain long noncontractile tails, and *Podoviridae* contain short noncontractile tails). However, at the genome level they can be diverse and share no DNA sequence similarity. The most abundant of these is the *Siphoviridae* phage, which is not limited to the marine environments but also to many other habitats [71,72].

More recently, an emerging focus has been placed on phages in deep-sea environments characterized by extreme conditions including the recently established Global Ocean Viromes 2.0 dataset [73]. This work greatly expands on the existing body of knowledge surrounding these largely unexplored extreme regions including those of hydrothermal vents, cold temperatures, low light, low oxygen, and high pressure. Interestingly, the broad composition seems to be similar to phage populations seen in other marine environments, with *Caudovirales* dominating [73,74]. The main difference between surface-layer and deep-sea habitats, however, is the abundance of temperate phages in the latter [75]. It is thought that with such low host cell diversity, a small number of temperate phages dominate the environment, and thus it is likely that they have a strong impact on the bacterial communities there. More studies are required to confirm their potential ecological impact within the two different habitats.

### 2.3. Bacteriophages in Soil

In comparison to marine environments, the soil virome remains relatively understudied [76]. Soil constitutes a significant proportion of the global biome and plays a key role in biogeochemical process including the turnover of carbon, nitrogen, and phosphorous (reviewed in [77]). Critical to these processes is a complex and diverse microbiome including bacteria, archaea, and fungi. These communities are distributed heterogeneously in soils such that microbial communities within bulk soil and plant roots can be vastly different in composition and abundance [78]. Collectively, they play crucial roles in the maintenance of soil quality and have tangible impacts on plant growth. Bacteria are amongst the most significantly represented microorganisms within the soil [79]. Given the nature of the phage-host relationship, it is likely bacteriophages exert an important influence by regulating bacterial population dynamics and facilitating horizontal gene transfer by transduction and transformation.

Difficulties associated with extraction of viruses from soil have traditionally hindered the investigation of phages in this environment. Studies exploring phage densities in a range of soil types at different geographical locations have used epifluorescence microscopy or transmission electron microscopy (TEM) to estimate VLP (virus-like particle) numbers [80,81,82]. Their number has been reported to be in the order of 10^9^ per g of dry soil, with variance limited to an order of magnitude across diverse soil types globally [83]. The virus-to-bacterium (VBR) ratio, however, appears to significantly differ depending on the soil type, where virus counts are 10- to 100-fold lower than bacteria in desert and agricultural soils, and 1000-fold higher than bacteria in Antarctic soil [83]. This contrasts to the more homogenous marine environment that shows a much narrower VBR ratio with an average of approximately 10-fold higher abundance of virus numbers as compared with those of bacteria [84]. The relative heterogeneity of soil is thought to account for the substantial variance seen in soil virome load, with moisture content, pH, and temperature being the key factors that control viral abundance and stability [83]. Furthermore, handling and processing of different environmental samples in the field and laboratory may also affect apparent VBR counts, and it is likely that strict standardization of methods between different studies may shed further light on the relative abundance of the soil virome.

The diversity of the virome in different soils seems to reasonably reflect the corresponding diversity of the bacteriome. A TEM-based study of six distinct soil types in Delaware demonstrated that, on average, tailed viruses were the most abundant (~80%) type in most soils [81]. This is in contrast to another TEM-based study in Scotland where tailed viruses only accounted for ~5% of the total viral population, whereas, soils were abundant in viruses with a small spherical morphology [82]. While conclusions on diversity have so far primarily been based on microscopy-based methods (TEM), more recent metagenomics-based approaches will be required to uncover the true diversity across varied soil types [85,86].

The ecological significance of phages in soil, as with viruses in other environments, probably lies in their ability to mediate bacterial growth rates and selectively impact the diversity of bacterial soil communities. It is not surprising that different phages play different roles in individual soil niches. Phages have been implicated in mediating biogeochemical properties of soil through the control of bacterial abundance and hence nutrient cycling capabilities [87], while phages located within the root-adjacent rhizosphere appear to influence the efficacy of symbioses between plant roots and bacteria [88,89]. Soil phages have also been applied in biocontrol protocols which is an elegant alternative to more traditional techniques for treating plant disease. This type of approach has already been successfully implemented in phage therapy to control the bacterial plant pathogen *Dickeya solani*, which is the cause of blackleg and rot in potato [90]. Ultimately, the development and standardization of methodologies for the extraction and investigation of viral assemblies from soils will continue to unravel new phenomena, and novel opportunities for the use of soil phages.

## 3. Bacteriophages in Artificial Environments

### 3.1. Bacteriophages in Wastewater Treatment Plants

The activated sludge wastewater treatment process is the largest biotechnological process in existence. It utilizes a selected bacterial community to reduce the levels of organic and inorganic compounds so that treated water no longer represents an environmental threat from eutrophication of rivers and lakes [91]. The biomass is organized distinctively as aggregates called flocs. Briefly, this process consists of the introduction of influent into an aeration basin where the wastewater is aerated, in order to promote microbial growth and aggregate formation. Then, this is passed into secondary clarifiers where the biomass flocs, now increased as a consequence of growth settle rapidly, and the liquid phase are removed. The key feature of the process is that most of the settled biomass is then recycled to the head of the plant. Since it contains the populations best suited to deal with the incoming influent, the activated sludge process is rapid and occupies a small footprint. Some of the biomass is discarded to maintain the biomass concentration at a predetermined level, and this control of sludge age is the main way the process is controlled.

Phages are highly abundant and diverse in activated sludge, with a predicted concentration ranging from 10^8^ to 10^9^ virus-like particles per ml of mixed liquor [92]. This high abundance of virus, mainly consisting of members of the *Siphoviridae* family, has received further support from a metagenomic study where 36% more viral DNA was found there as compared with soil, plant-associated, and other engineered systems [93]. Despite an understanding of the activated sludge process and the viruses involved, little research into phage communities had been conducted prior to 2011 [94]. With the advent of next-generation DNA sequencing (NGS) of isolated phages and metagenomics studies, this situation has improved [95,96,97,98,99,100,101,102,103,104,105,106]. It is now clear that the genomes of many bacteria present in activated sludge systems contain CRISPR-Cas regions, suggesting that these have in the past been infected by phage, and therefore their presence makes the problem of determining host/phage relationships in the absence of conventional ability to culture host cells, and consequently phage recovery [107].

Although it is still not clear what impact phages have on the activated sludge community, Brown et al. [106] hypothesized that phages are an important factor impacting their composition, and hence are likely to negatively affect plant performance. Evidence by [108] supported this, and suggested that phages may play an important role in performance deterioration of nitrifying bacteria in activated sludge. They used a lysogenic strain of *Nitrosospira multiformis*, whose genome was known to contain two prophages. By exposure to stress conditions such as low pH (an event inherently associated with the oxidation of NH_3_), high temperature, and exposure to toxic chemicals, the lytic cycle of these temperate prophages could be induced. Virion replication led to an increase in their abundances, a corresponding drop in the numbers of *N. multiformis*, and deterioration in rates of nitrification activity. Phages have also been held responsible for deterioration of the phosphorous removal capacity. Thus, it has been suggested that exposure of *Accumulibacter* to several stress factors induced the lytic cycle in prophages, known to occur in *Accumulibacter* genomes [107,109,110]. This led to an increase in VLP numbers, a decrease in the cell numbers of *Accumulibacter*, i.e., a phosphorous accumulating organism, and in the copy numbers of *ppk1*, the gene responsible for the synthesis of polyphosphate. Consequently, biomass phosphorus uptake rates and intracellular phosphate levels fell, and hence P removal capacity decreased. Barr et al. [111] also presented indirect evidence of phage lysis of *Accumulibacter* which lead to a decrease in plant performance.

Clarification of what impacts phages in activated sludge might have raised the question of whether they might provide a specific environmental control method for the problematic bacteria found in activated sludge [112]. Proliferation of some bacterial members can lead to the severe global operational problems of bulking and foaming caused mainly by filamentous bacteria [113,114,115], for which few effective treatment options exist [116]. The idea is to reduce the population levels of these below the threshold needed to sustain a bulking or a foaming event. Considerable interest has been shown in using phage therapy to control episodes of bulking (brought about by highly hydrophobic bacteria [117]). Phages lytic for most of these foaming bacteria have been isolated and their genomes sequenced [98,99,100]. Certainly, under lab conditions, these phages are highly effective in controlling foaming, but much still remains to be learned before these can be used with industrial scale plants [103,105]. For example, no phage has ever been isolated for some widespread foamers, and therefore whether their genomes contain defense cassettes [118] or CRISPR-Cas regions needs to be assessed.

### 3.2. Bacteriophages in Industrial Applications

Phages are a potential solution for the elimination of pathogens in the food production industry. Each year, foodborne hazards, predominantly bacterial, cause 420,000 deaths and an estimated 600 million cases of foodborne infections globally [119]. Phages are used currently in food processing in countries including Canada, Israel, and the USA to target pathogenic organisms such as the serious human pathogens *Listeria monocytogenes*, *Salmonella* serotypes, and *Escherichia coli* 0157:H7 [120]. Another potential application includes treating meats where treatment with phages was found to extend the shelf life of products such as pork and beef steaks as early as 1990 [121,122]. However, these studies were performed under laboratory conditions and later work by the same team found that a phage cocktail was ineffective in lysing targeted *Pseudomonas* strains, possibly reflecting a mismatch in phage/host specificity [121]. Additionally, the solid nature of the food product is significantly different from the broth medium where phage cocktails are commonly developed [123]. A liquid medium supports motility of both phage and bacterium, thus maximizing the likelihood of phage infection [124]. Increasing phage concentration may be one means of overcoming this obstacle. Multiple studies have reported a linear relationship between phage concentration and the inhibition of bacterial growth [125,126,127]. A 2016 study observed that an application of 10^5^ bacteriophage BPECO19 per *E. coli* O157:H7 bacterium completely inhibited growth within eight hours, while administration of 10^4^ phage particles required 72 h to similarly inhibit growth [127].

Phages occur naturally in the production of fermented foods including sauerkraut and dairy products such as cheese and yoghurt. Phages promote and enhance the production of sauerkraut by inhibiting undesirable bacterial species [128]. This is not the case with the dairy products where phages impede the lactic acid fermentation, in part because of the thermotolerance of some phages surviving pasteurization [122]. Phages that target *Lactobacillus helveticus*, which are used to produce cheese, can withstand the standard pasteurization temperature of 72 °C [129]. While this innate property may be beneficial in other applications, it is a hindrance in the production of fermented dairy products.

As reviewed in other literature [130], microbiologically induced corrosion of stainless-steel equipment arises as bacteria impact the kinetics of oxidation–reduction potential, thus increasing the rate of corrosion. This increase may occur through the formation of a biofilms or the production of hydrogen sulphide (H2S). Phages are being investigated widely in industry as a solution to microbiologically induced corrosion. Sulphate reducing bacteria, including members of the genus *Desulfovibrio*, are problematic in the petroleum industry as they produce H_2_S, which can then be oxidized to H_2_SO_4_ by chemolithotrophic bacteria [131]. In silico screening for prophage genes encoding endolysins or holins to be engineered into phages to target the problematic *Desulfovibrio* have been reported [131]. In vitro work found that phages were successful in inhibiting the growth of the biofilm producing *Stenotrophomonas maltophilia* [132]. As noted by a team utilizing a combination of two phages on sulphate-reducing bacteria that produce H_2_S, the extreme specificity of phages represents a challenge to finding such a phage [133].

### 3.3. Genetic Engineering Phages in the Laboratory

The potential applications of phages in natural and artificial environments may have certain limitations. However, with advances in molecular techniques, phages can be genetically modified in vitro to enhance current functions. Here, we discuss several recent approaches and applications of engineered phages for their use in environmental, industrial, and clinical areas. Continued exploration into phage genomics and engineering methods will help to advance their efficacy and function in downstream uses.

As pathogens continue to evolve by becoming resistant to antibiotics, the creation of effective and sensitive treatments is vital. Most naturally occurring phages display a narrow host range, infecting single or a few strains of a given species [6]. This can be problematic for phage therapy as it would be advantageous for a phage to be able to have a broader host range and infect multiple strains of the same genera. Research has been undertaken to alter host ranges of phages to either expand their infectivity or change its host from one strain to another which can lead to the creation of “personal therapeutics”, phages that have been engineered to treat a patient’s specific bacterial infection. Ando et al. [134] established a simple yet efficient yeast-based platform for the modification of phage host ranges by using common viral scaffolds and gap repair cloning to swap the tail fiber gene, *gp17*, between the two highly similar coliphages, T3 and T7 [134]. These phages were assembled in *Saccharomyces cerevisiae* resulting in the switching of host ranges without any decrease in lytic abilities. In another study, Mahichi et al. [135] determined it was possible to create a phage with an expanded host range to target multiple *E. coli* strains. The tail fiber genes, *gp37* and *gp38*, from a broad-spectrum phage, IP088, were integrated into to a narrow host range T2 phage by exploiting double-crossover homologous recombination [135]. This resulted in an expanded host range of the T2 phage to include both its original host range with the addition of the IP088 host range. Similarly, Marzari et al. [136], used the filamentous phage, Ike, to increase the host range of filamentous phage, fd, by the addition of a receptor-binding domain that encoded the detection of the bacterial cell wall receptor, N-pili. The recombinant phage, fd, was then able to infect *E. coli* strains with the N-pili. The fd phage has also been engineered to infect *Vibrio cholerae* in conjunction with the natural host *E. coli* [137]. This was achieved by fusing a minor coat gene *pIII* from the fd phage with a sequence of the *orfU* gene that encoded the N-terminal of another minor coat protein from another filamentous phage, CTXΦ [137]. The recombinant phage was successfully able to infect both *E. coli* and *V. cholerae*.

While having direct antimicrobial activity, phages can be genetically modified to enhance the mechanism of antibiotics or carry enzymes to enhance their natural abilities. Lu and Collins [138] engineered the phage M13mp18 to overexpress the repressor of a SOS DNA repair system. The overexpression of *lexA3* increased the efficacy of quinolone antibiotics, improved the activity of ampicillin and gentamicin antibiotics, reduced emergence of antibiotic resistance, and increased antibiotic-mediated killing of the *E. coli* strains that had already acquired resistance genes [138]. Aside from increasing antibiotic activity, phages can also be engineered to deliver other compounds including enzymes, which can aid in the dispersal of biofilm formations. The toxin pesticin, produced by *Yersinia pestis* to kill competing bacteria, pesticin, fused to the N-terminal of T4 phages lysozyme protein to expand its target to cells with pesitcin immunity [139].

Biofilms are complex microbial communities embedded in a matrix of extracellular polysaccharide substances (EPS). They are robust and difficult to degrade, causing persistent bacterial infections. To aid in the disruption of biofilms, the phage T7 was modified to express the enzyme, dispersin B (*dspB*), which is known to assist in the degradation of biofilms [140]. The engineered T7 phage encoding *dspB* was found to reduce cell counts of *E. coli* two orders of magnitude more than when compared to wildtype T7 [140]. Briers et al. [141], have been successful in generating artilysins, synthetic phage endolysins fused with lipopolysaccharide-destabilising peptides. Artilysins show promising results with decreased survival of *Salmonella* Typhimurium, *Pseudomonas aeruginosa*, and *Acinetobacter baumanii* counts when infected with the recombinant phages carrying artilysins [141].

Biosensors are detection tools of biological materials. An example of this is the creation of a reporter phage by inserting a fluorescent marker into a phage genome to produce fluorescence during infection of a specific bacterial host. Through the phage’s lytic life cycle and self-dosing ability, the marker will increase in concentration over time as the phage replicates, emitting a signal that can be easily detected. The green fluorescent protein (GFP) is a bioluminescent protein commonly used to measure gene expression and has several advantages including not requiring exogenous substrates to produce fluorescence, being small in size (~700 bp), having long lasting signal time, high stability, and low toxicity. Oda et al. [142], used GFP to develop a phage biosensor for the detection of enterohemorrhagic *E. coli* O157:H7. The GFP was fused to the C-terminus of the smaller outer capsid protein of the phage PP01 through double-crossover homologous recombination. Fluorescence could be detected within an hour of incubation using fluorescent microscopy [142]. For use in industry, a practical and portable detection method is vital. Vinay et al. [143], developed a “phagosensor” prototype using the HK620 phage to successfully detect *E. coli* and *Salmonella* sp. in water samples. The fluorescence gene, *gfpmut2*, was integrated into the phage genome and fluorescence could be easily measured by a portable flow cytometer to allow simple, onsite detection. This method was fast and sensitive, with detection of bacteria as few as 10 cells/mL of seawater with no prior enrichment [143]. The *luxCDE-luxAB* system has also been used extensively to create reporter phages for the detection of *Mycobacterium tuberculosis* [144], *S. typhimurium* [145], *E. coli* [146], *Listeria* [147], and *Bacillus anthracis* [148] through bioluminescence. For bioluminescence to occur, this system requires enzymatic activity of luciferase with the addition of a substrate, luciferin, for expression. Schofield et al. [149] successfully integrated *luxAB* into the *Y. pesttis* phage ΦA1122 and detected bioluminescence within 12 min after inoculation. While signal times and strengths depend on cell density, detection could occur in as little as 1000 *Y. pestis* cells within 60 min [149]. While sensitive, there were issues with detection of background signals, and further optimization is required to decrease both false positives and negatives.

Recently, the first case of using a genetically engineered phage for human treatment was reported [150]. The phage ZoeJ was modified to have its repressor gene deleted, increasing lytic efficiency. As a part of a cocktail with two other phages, modified ZoeJΔ45 was used in combination with antibiotics to successfully treat a chronic and life-threatening *M. abscessus* subsp. *Massiliense* infection in a 15-year-old cystic fibrosis patient. While no direct correlation between patient improvement and the use of phage treatment can be made, it is evident that reduced morbidity was seen, and often these chronic infections are associated with high morbidity and mortality. It is also noted that phage replication was seen in vivo over the course of treatment, particularly within the first week [150]. Employing phages, particularly those modified with additional advantages, for future treatment of chronic and antibiotic resistant infections should be encouraged. Further research to understand the biology of phage–host interactions and the optimization of gene editing techniques should continue for further advancement in phage genome editing.

### 3.4. Bacteriophage in Pharmaceutical Formulations

As with the formulation of any pharmaceutical agent into semi-solid dosage forms such as creams or ointments, it is important that phages are incorporated into the transfer vehicle so that there is homogeneity throughout the final product. This ensures consistent delivery of the medicament. An obstacle to the medicinal use of phages is that unlike pharmaceutical drugs, phages are large biological entities which rely on the integrity of their structures, for instance, their tails, for proper biological functioning. An acute example of the importance of the integrity of phage structures for therapeutic applications was seen in the recent PhagoBurn clinical trials in Europe [151], where poor therapeutic efficacy of the phages was reported. This may have resulted from physical damage to the viruses during preparation of the therapeutic dressings used.

To achieve delivery to the epithelia, phages can be formulated into aqueous solutions such as lotions, drops, and sprays; in viscous preparations such as hydrophilic gels; or semi-solid preparations such as creams, ointments, and pastes. The efficacy of formulation, stability and release of phages from semi-solid preparations has been shown in vitro [152]. Factors which have been shown to be important in stability are temperature and light exposure, such that optimal stability is achieved when phage formulations were stored in light protected containers at 4 °C [152]. Another factor of importance is the ionic nature of the semi-solid base. The overall electrostatic charge of phages changes with the pH of the environment [153], and phage capsids and tails can carry opposite charges [154,155]. Ionic polymers within creams may interact with phages through electrostatic forces. In experimental conditions, differential release of phage from creams of varying ionic nature was seen, with optimal release from nonionic formulations [156]. Formulations thicker than creams, such as ointments and pastes, may not be optimal for the delivery of phage for therapy. Components of pastes, for instance, may impart thickness to the final product, inhibiting the movement of phages formulated within [156].

The effect of preservatives in creams and ointments on phages is an area where little data exists. It has been suggested that some preservatives do affect phages adversely [157], and those which are acidic tend to have a more profound effect [158]. In an extensive study of the effect of preservatives on diverse types of phages, specific preservatives were found to significantly impact the efficacy of phages [156]. Apart from aqueous and semi-solid preparations, phages have been formulated into and shown to be successfully released from solid dosage forms such as pessaries, suppositories, and lozenges. These in vitro experiments demonstrated that phages were stable in these forms if stored protected from light at 4 °C [156].

In comparison to the therapeutic delivery of phages to the epithelia, systemic, and internal delivery of phages for treatment of sepsis and infection of internal organs and tissues offers greater hurdles. Animal models have demonstrated the capacity of phages to be delivered as injections for systemic sepsis infections [159]. Experiments have also begun to clarify the same immunological issues involved with therapeutic treatment using viruses [160,161]. While reports of successful application of phages via injection for treatment of such internal infection in humans have been relatively rare, these studies have been conducted [162,163,164]. With advocation for their potential to complement or substitute for antibiotics in therapy it is expected that there would be more clinical trials and diverse applications in the future [165,166].

There are challenges in the successful oral administration of phages for therapeutic delivery to the gut and for systemic absorption. As mentioned above, formulations in suppositories have been developed, with this delivery mode having the benefit of avoiding potential phage degradation by stomach acidity [152]. While some phages may be expected to survive the harsh gastric environment, formulation of lyophilized phages with a stomach acid-reducing drug such as a proton pump inhibitor or an H_2_ receptor antagonist may assist [167]. Furthermore, the technology to spray dry and microencapsulate phages for compression into tablets allows for significant protection against gastric fluid [168,169]. These dosage forms, therefore, offer realistic potential for gut and systemic delivery of these agents in a manner equivalent to standard pharmaceutical drugs.

## 4. Conclusions

In this review, we discuss the presence of bacteriophages in the natural and artificial environments. The natural environments explored include the human body, marine environments, and soil. The artificial environments included wastewater treatment systems, industrial applications, and laboratory-based techniques, which include genetic engineering of phages and the development of pharmaceutical products. Phages are present in all environments in coexistance with their bacterial hosts. Understanding phage lifecycles and their interaction with their corresponding host can be beneficial for reducing or eliminating recalcitrant bacterial populations, as well as understanding population dynamics. While there are numerous studies and reviews on phages in their natural environments, less attention has been given to the artificial environments. As further research into this field is conducted, it is likely that the impact of phages in various communities will be better understood. In turn, this knowledge can be exploited for a range of applications.
